# Trigonelline Alleviates Neuroinflammation in AAV‐SynA53T Parkinson's Disease Model Mice: Involvement of PI3K/AKT–NF‐κB Signaling and Gut Microbial‐Metabolic Remodeling

**DOI:** 10.1002/fsn3.72451

**Published:** 2026-10-06

**Authors:** Mozi Chen, Yiyao Dong, Jie Zheng, Yan Lin, Qiyao Li, Chuhua Wei, Linpei Zhuo, Yan He, Jing Sun, Feng Fu, Jiaming Liu

**Affiliations:** ^1^ School of Public Health Wenzhou Medical University Wenzhou Zhejiang China; ^2^ Department of Neurology The Second Affiliated Hospital of Wenzhou Medical University Wenzhou Zhejiang China; ^3^ Department of Neurology The First Affiliated Hospital of Wenzhou Medical University Wenzhou Zhejiang China; ^4^ School of Ophthalmology and Optometry and Eye Hospital Wenzhou Medical University Wenzhou Zhejiang China; ^5^ Department of Neurology Zhuji Affiliated Hospital of Wenzhou Medical University Zhuji Zhejiang China

**Keywords:** gut‐brain axis, multi‐omics integration, neuroinflammation, Parkinson's disease, PI3K/AKT–NF‐κB signaling, trigonelline

## Abstract

Trigonelline (TG), a major alkaloid in fenugreek seed and coffee, has anti‐inflammatory and antioxidant activities. We investigated whether TG ameliorates Parkinson's disease (PD)‐related synucleinopathy and whether these effects are associated with inflammatory signaling and gut microbial–metabolic alterations. Two‐sample Mendelian randomization (MR) was used to examine the relationship between genetically predicted circulating TG and α‐synuclein. In vivo, AAV‐SynA53T C57BL/6J mice received oral TG (5 mg/kg/day) for 30 days, followed by assessment of motor behavior, pSer129‐α‐syn pathology, dopaminergic markers, inflammatory readouts, intestinal barrier proteins, 16S rRNA profiles, and colon‐content metabolomics. MR supported a potential protective association between genetically predicted serum TG and reduced α‐synuclein. TG improved motor performance, reduced midbrain pSer129‐α‐syn signals, preserved dopaminergic markers, and attenuated inflammatory responses, accompanied by modulation of PI3K/AKT and NF‐κB signaling. TG also improved intestinal barrier‐associated features and altered the gut microbiota and metabolomic profile. A metabolomic feature tentatively annotated as Ganolucidic acid B was increased following TG treatment and correlated with several taxa enriched in the TG group; conditional network pharmacology suggested that its predicted targets were enriched in PD‐relevant inflammatory and PI3K–AKT‐related pathways. Overall, TG may ameliorate synucleinopathy‐associated phenotypes in AAV‐SynA53T mice, accompanied by attenuated inflammation and gut microbiota–metabolism remodeling. Further targeted metabolomic validation and causal intervention studies are warranted to clarify the contributions of specific metabolites and gut‐associated mechanisms.

## Graphical abstract

Trigonelline alleviated motor deficits, α‐synuclein‐associated pathology, and neuroinflammatory changes in AAV‐SynA53T mice. These effects were accompanied by reduced PI3K/AKT–NF‐κB signaling activity, improved intestinal barrier‐associated features, and gut microbiota–metabolome remodeling.

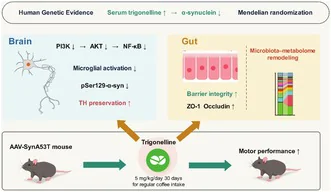

## 1 Introduction

Parkinson's disease (PD) is a progressive neurodegenerative disorder pathologically defined by the accumulation of misfolded α‐synuclein (α‐syn) into Lewy bodies and the subsequent loss of dopaminergic neurons in the substantia nigra pars compacta (SNpc) (Ren and Chen 2020; Spillantini et al. 1997). While motor deficits—such as bradykinesia, rigidity, and tremor—are the clinical hallmarks of PD, the disease is increasingly recognized as a systemic condition involving complex non‐motor symptoms and pervasive neuroinflammation (Bloem et al. 2021; Grayson 2016). Misfolded α‐syn functions as a damage‐associated molecular pattern (DAMP), triggering microglia into a chronic pro‐inflammatory state. This activation creates a self‐perpetuating neurotoxic loop: activated microglia release pro‐inflammatory cytokines (e.g., TNF‐α, IL‐1β) and reactive oxygen species (ROS) via pathways such as NF‐κB, which exacerbates neuronal death and further impairs the microglial phagocytosis of α‐syn aggregates (Isik et al. 2023; So et al. 2024).

Beyond the central nervous system (CNS), the “gut‐brain axis” has emerged as a critical frontier in understanding PD etiology. The Braak hypothesis suggests that PD pathology may originate in the enteric nervous system (ENS) and propagate to the brain via the vagus nerve in a prion‐like manner (Braak et al. 2003). Gastrointestinal dysfunction and gut dysbiosis often precede motor symptoms in PD. Altered gut microbiota may impair the intestinal barrier and promote systemic inflammation, potentially contributing to neurodegeneration (Chen et al. 2025; Oliver et al. 2025). Despite these insights, current therapeutic strategies, primarily relying on dopaminergic replacement (e.g., levodopa), remain purely symptomatic and fail to arrest the underlying protein aggregation or neuroinflammatory cascades. Consequently, there is an urgent need for disease‐modifying therapies that can simultaneously target central neuroinflammation and peripheral gut dysbiosis (Lopez‐Diego and Weiner 2008; Mattson 2004; Mestre et al. 2009).

Dietary phytochemicals offer a promising reservoir for such multi‐target agents. Trigonelline (TG), a pyridine alkaloid abundant in coffee beans and fenugreek seeds, has attracted attention for its neuroprotective potential (Socała et al. 2020; Zhou et al. 2022). Epidemiological studies suggest an inverse correlation between coffee consumption and PD risk, with TG identified as a key bioactive contributor (Makiso et al. 2024). Preclinical research indicates that TG exerts antioxidant and anti‐inflammatory effects; for instance, it has been shown to mitigate lipopolysaccharide (LPS)‐induced cognitive impairment by downregulating TLR4/NF‐κB signaling (Khalili et al. 2018). However, whether TG can effectively mitigate α‐synucleinopathy in a PD model, and the precise molecular mechanisms linking its central effects to its peripheral modulation of the gut microbiome, remain to be elucidated.

In this study, we evaluated the neuroprotective effects of TG in an AAV‐A53T α‐synuclein overexpression mouse model and explored whether these effects were associated with inflammatory signaling and gut microbial‐metabolic remodeling. We employed an integrative approach combining Mendelian randomization analysis, in vivo experimental validation, 16S rRNA sequencing, untargeted metabolomics, and network pharmacology. Rather than establishing a definitive causal gut‐brain axis pathway, our study was designed to identify potential associations among TG treatment, neuroinflammation, microbial alterations, candidate metabolites, and PI3K/AKT–NF‐κB signaling.

## 2 Materials and Methods

### 2.1 Data Sources and MR Analysis

We performed two‐sample MR analysis using summary‐level data from large‐scale, open‐access genome‐wide association study (GWAS) databases. This approach relies on three core assumptions validated through sensitivity analyses: (i) strong association between instrumental variables (IVs) and target exposure (*F*‐statistic > 10); (ii) exclusion of pleiotropic pathways connecting IVs and outcome independent of exposure; (iii) absence of confounding factors influencing IV‐outcome relationships. Genetic instruments for serum trigonelline (GWAS accession ID: ebi‐a‐GCST90266095, *n* = 4960) and α‐synuclein levels (GWAS accession ID: GCST90240258, *n* = 3301) were derived from publicly available databases, including the NHGRI‐EBI GWAS catalog. Genetic analyses were conducted using the TwoSampleMR package (v0.6.1) in the R environment. Instrumental variables meeting genome‐wide significance (*p* < 5 × 10^−6^) were selected through phased screening: (1) LD pruning (r^2^ < 0.001, 10,000 kb window); (2) exclusion of palindromic SNPs (Auton et al. 2015); (3) *F*‐statistic > 10 threshold for strength validation (Yao et al. 2023). The specific SNPs used as instrumental variables and their corresponding F‐statistics are provided in Table S1. The IVW method served as the primary analysis, supplemented by four pleiotropy‐robust methods (WME, WM, MR‐Egger, ML). Comprehensive sensitivity validation included: Cochran's Q test for heterogeneity (*p* > 0.05); MR‐Egger intercept/MR‐PRESSO for horizontal pleiotropy (*p* > 0.05); leave‐one‐out stability assessment. Statistically significant associations (*p* < 0.05) were interpreted through odds ratios (OR) with 95% CIs, where OR > 1 indicates positive association and CI exclusion of 1 confirms significance.

### 2.2 Target Identification and Prioritization

The SMILES structure of Ganolucidic acid B (CID 20055994) was retrieved from PubChem for systematic target prediction across three databases: SuperPred, SwissTargetPrediction, and TargetNet. PD‐associated targets were simultaneously curated from GeneCards using relevance scores > 10.

### 2.3 PPI Network Construction

Protein–protein interactions were analyzed using STRING v12.0 with species restricted to *Homo sapiens* and high‐confidence interaction thresholds (score > 0.7). The resultant network was imported into Cytoscape v3.10.3 for visualization, where edge thickness corresponded to interaction confidence scores.

### 2.4 Hub Gene Identification

Network topology analysis was performed using the CytoHubba plugin, which ranks nodes by three centrality metrics (Chang et al. 2022): Degree, Maximal Clique Centrality (MCC); Maximum Neighborhood Component (MNC). The top 15 nodes from each metric category were intersected through Venn diagram analysis.

### 2.5 Functional Enrichment Analysis

Gene Ontology (BP/CC/MF) and KEGG pathway annotations were generated through the DAVID database. Enrichment results were visualized via clusterProfiler v4.0 with ggplot2, focusing on neuroinflammation‐related pathways that showed consensus activation patterns across three independent algorithm implementations.

### 2.6 Animals and Virus Injections

C57BL/6J mice (Zhejiang Vital River Laboratory Animal Technology Co. Ltd., China) were maintained under standardized conditions (12 h light/dark cycle; 22°C ± 2°C; 60% ± 5% humidity) with ad libitum access to food/water. Conditional expression of recombinant AAV‐α‐Syn‐FLAG virus was achieved by stereotaxic injection into Substantia Nigra pars Compacta (SNc). About 0.2 μL/side α‐Syn virus and the control (AAV‐FLAG virus) were bilaterally injected into SNc (AP, −3.1 mm; ML, ±1.24 mm; DV, −4.24 mm). Injections were performed at 0.02 μL/min with 5‐min post‐injection stabilization. Three experimental groups were established: FLAG (AAV‐FLAG virus + saline gavage, *n* = 8), SynA53T (AAV‐α‐Syn‐FLAG + saline gavage, *n* = 8) and Trigonelline (AAV‐α‐Syn‐FLAG +5 mg/kg TG gavage, *n* = 8). Daily oral administrations commenced 2 weeks post‐surgery and continued for 30 consecutive days. The TG dosage (5 mg/kg/day) and administration duration (30 days) were determined based on previous research (Aktar et al. 2024), which confirmed this dose as effective and non‐toxic in mice models. Control groups received equivalent‐volume saline following identical schedules. At the end of the experiment, mice were deeply anesthetized with 1% sodium pentobarbital (50 mg/kg, i.p.) and transcardially perfused with ice‐cold physiological saline, followed by 4% paraformaldehyde (PFA). All procedures were approved by the Animal Ethics Committee of Wenzhou Medical University, following National Institutes of Health (NIH) guidelines.

### 2.7 Pole Test

The pole test was performed to assess bradykinesia and motor coordination in mice, as commonly used in behavioral assessment of rodent models of Parkinson's disease (Sheta et al. 2024). The apparatus consists of a 50‐cm vertical pole (1 cm diameter) with a textured surface. During testing, animals were positioned head‐downward at the apex, with descent latency (forelimb contact with the base platform) recorded digitally. Following single‐session acclimatization, three consecutive trials were averaged as the final score using automated tracking software.

### 2.8 Rotarod Test

Rodents underwent two‐phase motor coordination evaluation using an accelerated rotating rod system (Brooks and Dunnett 2009). Acclimatization protocol: 10 rpm for 5 min/day across two consecutive days. Formal testing consisted of three trials at 40 rpm acceleration mode (5‐min cutoff), with fall latency recorded by infrared sensors. Mean latency from three consecutive trials served as primary endpoint.

### 2.9 Balance Beam Test

Motor coordination was quantified on a 1 m‐long elevated beam (1 cm width) with safety platforms (Bidgood et al. 2024). Trials commenced with rodent placement at the beam origin, recording both traversal duration (nose contact with terminal platform). Three validated trials were averaged using automated tracking.

### 2.10 Immunofluorescence

Immunofluorescence staining of paraffin‐embedded tissue sections was performed according to previously described protocols with minor modifications (Zaqout et al. 2020). Freshly dissected mouse brains and colons underwent 24‐h fixation in 4% paraformaldehyde followed by standard paraffin processing: graded ethanol dehydration, xylene clearing, and paraffin embedding. Tissue sections (5‐μm thickness) were baked at 65°C for 2 h prior to staining. Antigen retrieval was performed in citrate buffer (pH 6.0) using microwave heating (95°C, 5 min). After cooling and permeabilization with 0.3% Triton X‐100 (15 min), sections were blocked with 5% BSA (1 h, 37°C) before incubation with anti‐α‐Syn antibody (1:200; Bioworld, MN, USA), anti‐Iba‐1 antibody (1:200, Abcam, Cambridge, UK), anti‐CD11b antibody (1:200, Bioworld, Louis Park, USA), anti‐FLAG antibody (1:200, Huaan Biotechnology, China), anti‐ZO‐1 antibody (1:200, Santa Cruz Biotechnology, USA), anti‐Occludin antibody (1:200, Proteintech Group, USA) at 4°C overnight. Secondary antibody conjugation (AF488‐labeled Goat Anti‐Rabbit IgG (H + L), 1:200 and Cy3, Goat Anti‐Rat IgG (H + L), 1:200) was followed by DAPI counterstaining. Coverslipped sections were imaged using laser scanning confocal microscopy (Leica TCS SP8) with standardized acquisition parameters.

### 2.11 Immunohistochemistry

Immunohistochemistry was performed on paraffin‐embedded tissue sections using chromogenic detection with minor modifications from established protocols (Muniz Partida and Walters 2023; Uhlen et al. 2016). Baking at 65°C for 2 h, xylene clearing (30 min), and graded alcohol rehydration followed. After heat‐mediated antigen retrieval, endogenous peroxidase activity was blocked with 3% H_2_O_2_ treatment (15 min) followed by PBS washing. Sections were blocked with 5% BSA (30 min, RT) before incubation with anti‐TH antibody (1:500, abcam, Cambridge, UK) at 4°C overnight. After rewarming (37°C, 30 min) and PBS rinsing, sections were treated with HRP‐conjugated secondary antibody (RT, 30 min). DAB substrate was applied for chromogenic development with hematoxylin counterstaining. Finally, dehydrated sections were mounted and examined under light microscopy for brown‐stained TH neurons.

### 2.12 Western Blot

Western blotting and densitometric quantification were performed according to recommended quantitative Western blot workflows (Pillai‐Kastoori et al. 2020). Tissue homogenates were centrifuged at 12,000 × g for 15 min to collect supernatants. Protein concentrations were determined using a BCA assay kit (Pierce). Protein samples (30 μg/lane) were heated at 95°C for 5 min, separated by 10% SDS‐PAGE, and electrophoretically transferred onto PVDF membranes. After blocking with 5% non‐fat milk for 1 h at RT, membranes were incubated with the p‐Syn (1: 1000, Bioworld, Minnesota, USA), TH (1: 1000, Bioworld, Minnesota, USA), phospho‐NFκB‐p65 (1: 1000, Bioworld, Minnesota, USA), NFκB‐p65 (1: 1000, Bioworld, Minnesota, USA), P‐AKT (1: 1000, Bioworld, Minnesota, USA), AKT (1: 1000, Bioworld, Minnesota, USA), COX2 (1: 1000, Affinity Biosciences), IL‐1β (1: 1000, Affinity Biosciences), Phospho‐PI3K (1: 1000, Affinity Biosciences), PI3K (1: 1000, Affinity Biosciences), TNF‐α (1: 1000, Huaan Biotechnology, China) at 4°C overnight. HRP‐conjugated secondary antibodies (1: 5000) were applied for 1 h at RT. Protein bands were visualized using ECL substrate and quantified by ImageJ software (NIH) with β‐actin (ET1702‐67, Huabio, 1: 4000) as loading control.

### 2.13 RT‐qPCR

Total RNA was extracted from tissue samples using TRIzol reagent (Invitrogen, catalog no. 15596026) following the manufacturer's instructions. RNA concentration and purity were assessed spectrophotometrically, and first‐strand cDNA was synthesized from 0.5–1 μg total RNA using a commercial reverse transcription kit (Takara, catalog no. RR047A) according to the supplier's protocol. qPCR reactions were performed on an ABI 7300 real‐time PCR system (Applied Biosystems) using SYBR Premix Ex Taq II. Reactions were assembled in 20 μL volumes and run in technical triplicates; no‐template controls were included to monitor contamination. Cycling conditions were applied as recommended by the reagent manufacturer and concluded with a melt‐curve analysis to verify product specificity. RT‐qPCR procedures and reporting were guided by the updated MIQE recommendations (Bustin et al. 2025). Relative expression levels of Il1b, Il6, and Nfkb1 were calculated using the 2^−ΔΔCt^ method with Actin as the endogenous control. Primer sequences are provided in Table S2.

### 2.14 16s rRNA Sequencing and Data Analysis

Colon content samples (200–300 mg) were immediately frozen in liquid nitrogen for 16S rRNA sequencing. Microbial DNA was extracted using the QIAamp DNA Stool Mini Kit (Qiagen). The V3‐V4 hypervariable regions of bacterial 16S rRNA genes were amplified with primers 338F (5′‐ACTCCTACGGGAGGCAGCAG‐3′) and 806R (5′‐GGACTACHVGGGTWTCTAAT‐3′) using the following thermal cycling protocol: 95°C for 3 min; 35 cycles of 95°C for 30 s, 55°C for 30 s, and 72°C for 45 s; final extension at 72°C for 10 min. Purified amplicons were quantified with Qubit 3.0 (Thermo Fisher) and sequenced on Illumina MiSeq platform (2 × 250 bp paired‐end). Raw reads were quality‐filtered using Trimmomatic v0.39 (SLIDINGWINDOW:4:20 MINLEN:200) and assembled with FLASH v1.2.11. Amplicon sequence variants (ASVs) were inferred using DADA2 pipeline. Chimeric sequences were removed using the Silva reference database v138. Taxonomic classification was performed using RDP Classifier (confidence threshold 0.8) against the Greengenes database v13_5. Alpha diversity indices (Ace, Shannon, Simpson) and beta diversity (Bray‐Curtis PCoA) were calculated with QIIME2. Differential taxa between groups were identified through LEfSe analysis (LDA score > 2.5, Kruskal‐Wallis *p* < 0.05). Because the omics analyses were designed to capture TG‐responsive changes under SynA53T pathology, only colon content samples from the SynA53T and TG groups were subjected to 16S rRNA sequencing, and the FLAG group was not included. The 16S rRNA sequencing workflow and methodological reporting were guided by recent standards for environmental and non‐human host‐associated microbiome studies (Bolyen et al. 2019; Kelliher et al. 2025).

### 2.15 Metabolite Analysis

Colon content samples were extracted for UHPLC–MS/MS analysis according to a previously described method (Feng et al. 2024). Briefly, ~100 mg of colon content was mixed with 800 μL methanol: water (4:1, v/v) containing L‐2‐chlorophenylalanine (0.02 mg/mL), homogenized (−10°C, 50 Hz, 6 min), ultrasonicated (5°C, 40 kHz, 30 min), incubated (−20°C, 30 min), and centrifuged (13,000 × g, 4°C, 15 min); the supernatant was collected for analysis. LC–MS/MS was performed on a UHPLC–Orbitrap Exploris 240 system (Thermo Fisher Scientific) with an HSS T3 column (100 × 2.1 mm, 1.8 μm; Waters) using mobile phase A (water/acetonitrile, 95:5, v/v, 0.1% formic acid) and mobile phase B (acetonitrile/isopropanol/water, 47.5:47.5:5, v/v/v, 0.1% formic acid) at 0.40 mL/min (3 μL injection; 40°C). Data were acquired in positive/negative ion modes (full scan m/z 70–1050 with data‐dependent MS/MS; stepped collision energy 20/40/60 eV). Raw data were processed in Progenesis QI (Waters) for peak picking and alignment, and metabolites were annotated against HMDB, Metlin, and the Majorbio database; the data matrix was further analyzed on the Majorbio cloud platform. Differential metabolites were defined using VIP > 1 and *p* < 0.05.

### 2.16 Statistical Analysis

The data were expressed as Mean ± SEM and analyzed by SPSS 22.0 software. The significance test was conducted by *t*‐test and one‐way ANOVA, and the difference between multiple groups was measured by Dunnett's multiple comparisons test. The results of 16 s rRNA were analyzed by Kruskal‐Wallis + Dunn's post hoc (Benjamini‐Hochberg correction) test. *p* < 0.05 was statistically significant.

## 3 Results

### 3.1 MR Analysis of Trigonelline and α‐Synuclein

To establish a causal foundation for the potential neuroprotective effects of TG, we employed a two‐sample Mendelian randomization (MR) approach to investigate the relationship between genetically predicted serum TG levels and α‐synuclein pathology. The primary analysis using the Inverse Variance Weighted (IVW) method revealed a significant protective causal association, indicating that higher genetically predicted TG levels are associated with reduced α‐synuclein levels (OR = 0.858, 95% CI: 0.753–0.977, *p* = 0.021; Figure S1A). Importantly, this finding was corroborated by the Weighted Median method, which also demonstrated a significant protective effect (OR = 0.818, 95% CI: 0.696–0.962, *p* = 0.015), suggesting that the causal estimate is robust even if up to 50% of the genetic variants are invalid instruments. The scatter plot further visualized this consistent negative correlation across the selected instrumental variables (Figure S1B).

To ensure the reliability of these estimates, we conducted comprehensive sensitivity analyses. We found no evidence of significant heterogeneity among the instrumental variables in either the IVW or MR‐Egger analyses (Cochran's Q test, *p* > 0.05), implying a consistent effect direction across different genetic loci. Furthermore, horizontal pleiotropy was ruled out, as indicated by the non‐significant intercept in the MR‐Egger regression and the results of the MR‐PRESSO global test (*p* > 0.05). Finally, the “leave‐one‐out” sensitivity analysis demonstrated that the observed causal association was not driven by any individual single‐nucleotide polymorphism (SNP), as the omission of individual variants did not alter the overall statistical significance (Figure S1C). Collectively, these genetic epidemiological data provide strong evidence supporting a potential causal role for TG in mitigating α‐synuclein accumulation.

### 3.2 Trigonelline Improves Motor Performance and Attenuates Nigrostriatal Pathology in SynA53T Mice

To evaluate the therapeutic potential of TG in vivo, we utilized an AAV‐mediated SynA53T overexpression mouse model. Following a 14‐day viral expression period, mice were administered TG (5 mg/kg) or saline daily for 30 days prior to behavioral assessment (Figure 1A). SynA53T mice exhibited profound motor deficits compared to the FLAG‐control group, characterized by impaired coordination and bradykinesia. However, TG treatment significantly ameliorated these motor dysfunctions. Specifically, TG‐treated mice showed a marked reduction in traversal time on the narrow beam test (Figure 1B) and a significant restoration of motor endurance in the rotarod test, as evidenced by prolonged latency to fall (Figure 1C). Similarly, in the pole test, TG treatment effectively reversed the SynA53T‐induced delay in crawling time (Figure 1D), collectively indicating that systemic TG administration preserves motor function in a PD mouse model.

**FIGURE 1:**
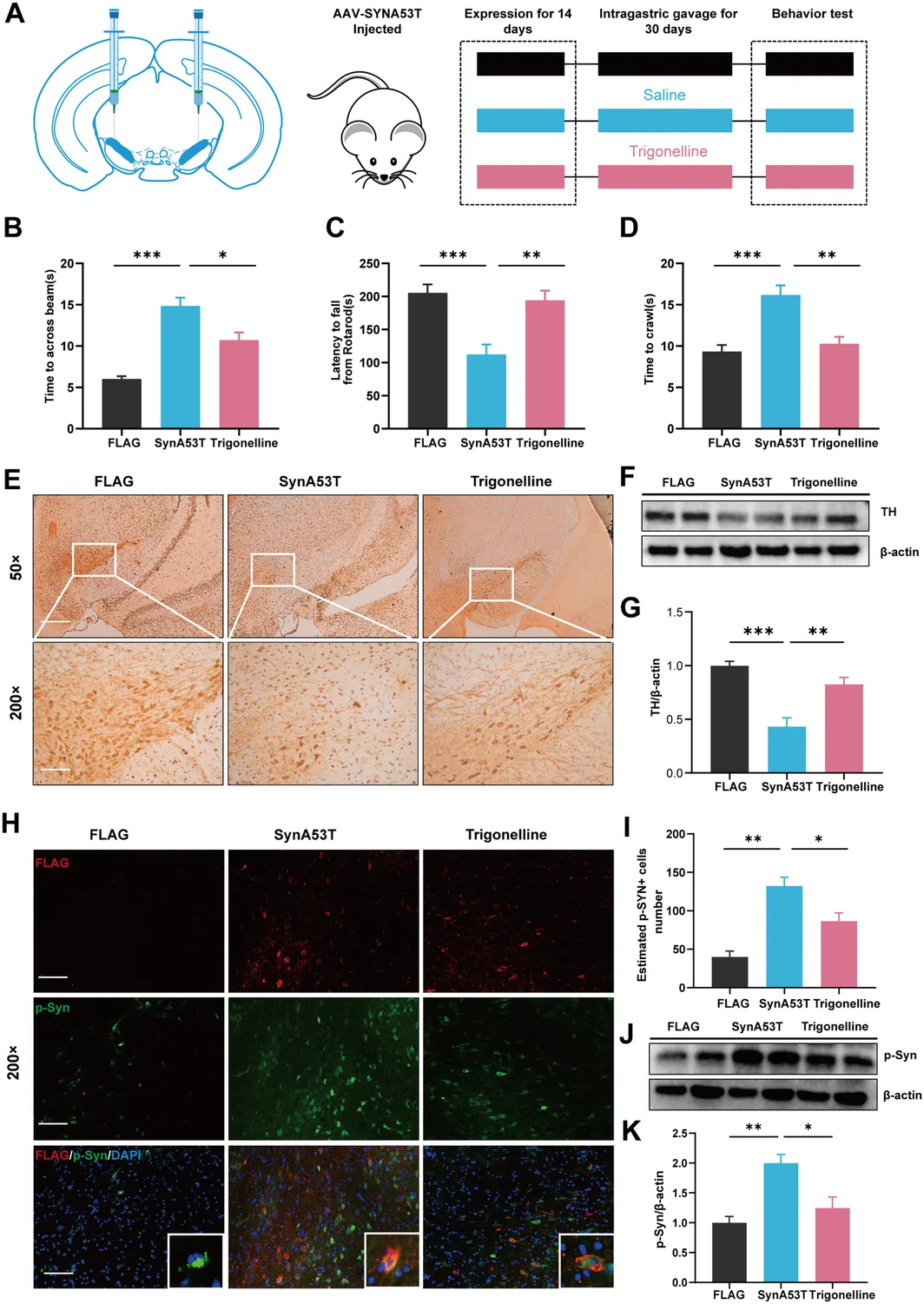
Trigonelline alleviates motor deficits and α‐synuclein pathology in AAV‐SynA53T mice. (A) Experimental timeline of AAV‐SynA53T induction and trigonelline treatment. (B–D) Behavioral assessments including beam test, rotarod, and pole test. (E) Immunohistochemical detection of TH expression in substantia nigra. Scale bars: 400 μm (upper panels); 100 μm (lower panels). (F, G) Western blot and quantification of TH protein levels. (H) Immunofluorescence of phosphorylated α‐synuclein (p‐α‐Syn, Ser129) and FLAG‐tagged A53T expression. Scale bar: 100 μm. (I) Quantification of the number of p‐α‐Syn positive neurons. (J, K) Western blot and quantification of p‐α‐Syn expression. Data are presented as mean ± SEM. Statistical significance: *p < 0.05, **p < 0.01, ***p < 0.001. TH, tyrosine hydroxylase; p‐α‐Syn, phosphorylated α‐synuclein; SN, substantia nigra.

We next investigated whether this functional recovery corresponded to the preservation of the nigrostriatal dopaminergic system. Immunohistochemical analysis of tyrosine hydroxylase (TH) in the substantia nigra pars compacta (SNpc) revealed a substantial loss of dopaminergic neurons in the SynA53T group. Notably, TG administration significantly attenuated this neuronal loss, preserving the structural integrity of the SNpc (Figure 1E). These histological findings were corroborated by Western blot analysis, which demonstrated that the SynA53T‐induced downregulation of TH protein levels was significantly reversed by TG treatment (Figure 1F,G), confirming the neuroprotective efficacy of TG against α‐synuclein‐driven dopaminergic degeneration.

Given that the accumulation of phosphorylated α‐synuclein at Serine 129 (p‐α‐Syn) is a pathological hallmark of PD that drives neurotoxicity, we examined the effect of TG on α‐synuclein aggregation. Immunofluorescence staining showed widespread accumulation of p‐α‐Syn (Ser129) co‐localized with FLAG‐tagged A53T expression in the SNpc of model mice. Strikingly, TG treatment markedly reduced the burden of pathological aggregates, as indicated by a significant decrease in the number of p‐α‐Syn‐positive cells (Figure 1H,I). Consistent with the imaging data, Western blot analysis of nigral lysates confirmed a significant reduction in p‐α‐Syn protein levels in the TG‐treated group compared to vehicle‐treated SynA53T mice (Figure 1J,K). Taken together, these data suggest that TG exerts its neuroprotective effects by suppressing the pathological accumulation of phosphorylated α‐synuclein, thereby attenuating dopaminergic neuron loss and alleviating motor deficits.

### 3.3 Trigonelline Attenuates Microglial Activation and Pro‐Inflammatory Signaling via the AKT/NF‐κB/TNF‐α Axis

In an effort to elucidate the mechanism by which TG exerts neuroprotection, we investigated the status of microglial activation and the associated inflammatory signaling cascades in the substantia nigra (SN) of AAV‐SynA53T mice. Immunofluorescence analysis revealed that SynA53T overexpression triggered robust microgliosis, characterized by significantly elevated immunoreactivity for the microglial markers Iba‐1 and CD11b compared to the FLAG control group (Figure 2A,B). Morphologically, microglia in the SynA53T group exhibited a hypertrophic, ameboid phenotype indicative of an activated state, whereas TG treatment markedly suppressed these morphological alterations and reduced the fluorescence intensity of microglial markers, suggesting a reversion toward a quiescent state. We next quantified the expression of downstream inflammatory mediators to corroborate the histological findings. Western blot analysis demonstrated that SynA53T injection significantly upregulated the protein levels of key pro‐inflammatory effectors, including COX‐2, TNF‐α, and IL‐1β, in the SN. Notably, TG administration significantly attenuated the increases in these inflammatory proteins (Figure 2C–F). To further examine the molecular signaling underlying this anti‐inflammatory effect, we assessed the PI3K/AKT/NF‐κB axis, a critical pathway regulating microglial activation. SynA53T mice exhibited hyperactivation of this pathway, as evidenced by increased phosphorylation ratios of PI3K (p‐PI3K/PI3K) and AKT (p‐AKT/AKT), together with increased activation of the NF‐κB subunit p65 (p‐p65/p65) (Figure 2G–J). In contrast, TG treatment significantly reduced the phosphorylation of PI3K, AKT, and p65, restoring these signaling changes toward control levels. Consistent with the protein‐level findings, quantitative PCR analysis revealed significantly increased mRNA levels of Il1b, Il6, and Nfkb1 in the SynA53T group, whereas TG administration markedly suppressed these transcriptional changes (Figure 2K–M). Collectively, these data indicate that TG‐mediated attenuation of SynA53T‐induced neuroinflammation was accompanied by reduced PI3K/AKT signaling activity and suppressed NF‐κB‐associated inflammatory responses.

**FIGURE 2:**
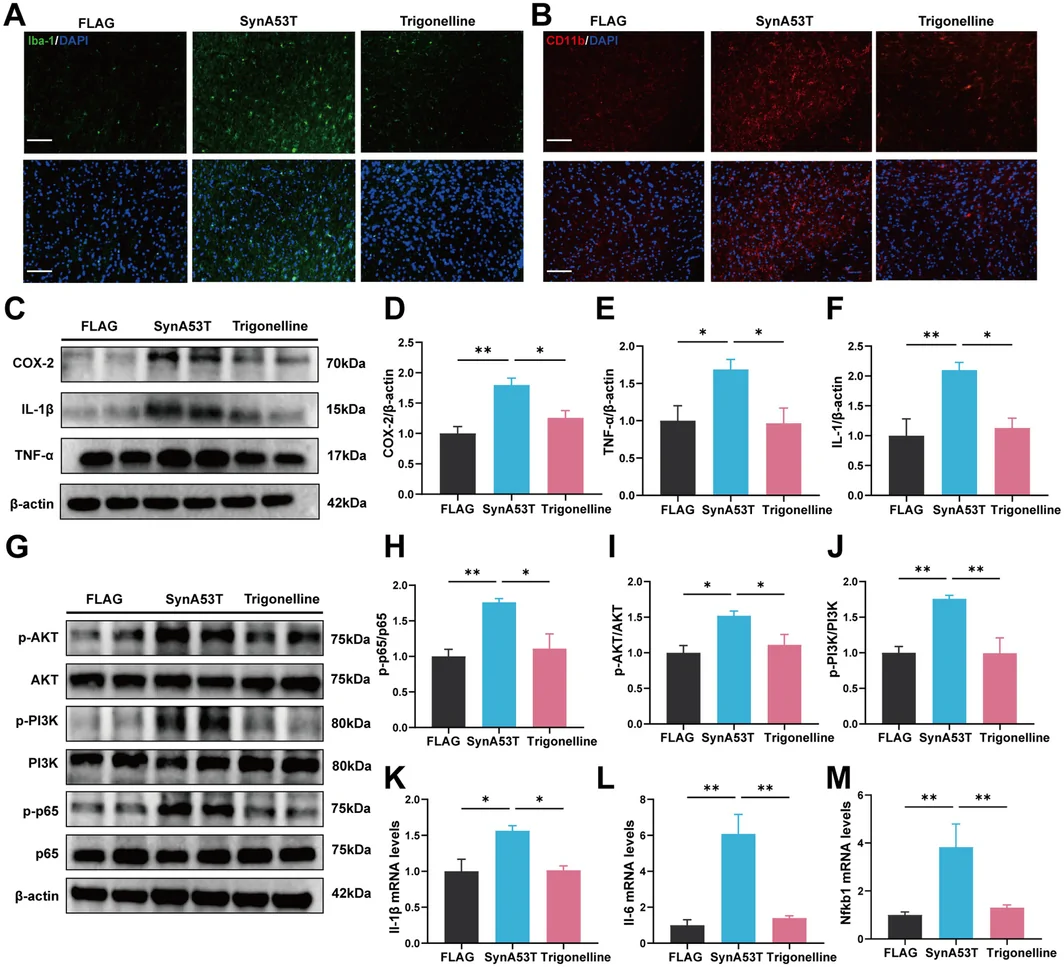
Trigonelline suppresses microglial activation and AKT/NF‐κB/TNF‐α signaling in substantia nigra of SynA53T mice. (A) Immunofluorescence staining for Iba‐1. Scale bar: 100 μm. (B) Immunofluorescence staining for CD11b. Scale bar: 100 μm. (C–F) Western blot and quantification of COX‐2, TNF‐α, and IL‐1β expression in the substantia nigra. (G–J) Western blot evaluation and quantification of p‐p65/p65, p‐AKT/AKT, and p‐PI3K/PI3K ratios. (K–M) qPCR analysis of Il1b, Il6, and Nfkb1 mRNA levels. Data are presented as mean ± SEM. Statistical significance: *p < 0.05, **p < 0.01, ***p < 0.001. Iba‐1, ionized calcium‐binding adapter molecule 1; TNF‐α, tumor necrosis factor‐alpha; AKT, protein kinase B; NF‐κB, nuclear factor kappa‐light‐chain‐enhancer of activated B cells; COX‐2, cyclooxygenase‐2; IL‐1β, interleukin‐1 beta; IL‐6, interleukin‐6; p‐PI3K, phosphorylated phosphatidylinositol 3‐kinase.

### 3.4 Trigonelline Attenuates Colonic Inflammation, Reduces Enteric p‐α‐Syn Accumulation, and Restores Barrier Integrity

Given that α‐synuclein pathology has been shown to propagate from the central nervous system to the periphery in the AAV‐SynA53T model (McFleder et al. 2023), we investigated whether TG could exert protective effects on the enteric environment. We first assessed colonic morphology using H&E staining. The SynA53T group exhibited marked mucosal atrophy, characterized by a significant depletion of goblet cells, reduced glandular depth, and disorganized epithelial architecture. In contrast, TG treatment effectively preserved mucosal integrity, maintaining goblet cell density and crypt structure comparable to the control group (Figure 3A–C). To determine if these morphological improvements translated to functional barrier recovery, we examined the expression of the tight junction proteins ZO‐1 and Occludin. In SynA53T mice, immunofluorescence analysis revealed a fragmented and diminished distribution of both proteins along the apical–lateral membranes, indicating compromised barrier integrity. TG administration reversed these defects, restoring the continuous, belt‐like organization of ZO‐1 and Occludin and significantly enhancing their fluorescence intensity (Figure 3D–F). We next characterized the local immune landscape and pathological burden. Staining for Iba‐1 and CD11b demonstrated a robust infiltration of myeloid cells (macrophages) within the colonic lamina propria of SynA53T mice, indicative of a severe local inflammatory response. TG treatment significantly attenuated this recruitment, reducing the density of Iba‐1+ and CD11b+ cells (Figure 3G–J). Concurrently, we evaluated the enteric accumulation of phosphorylated α‐synuclein (p‐α‐syn), a hallmark of PD pathology. While SynA53T mice displayed prominent p‐α‐syn immunoreactivity in the colon, TG‐treated mice showed visibly reduced p‐α‐syn signals (Figure 3K), suggesting that TG not only suppresses inflammation but may also alleviate the peripheral burden of α‐synuclein. Finally, to elucidate the molecular basis of the observed anti‐inflammatory effects, we quantified key inflammatory mediators in colonic tissue. Western blot analysis showed that SynA53T overexpression significantly increased the expression of COX‐2 and TNF‐α. TG treatment significantly suppressed the expression of both inflammatory mediators (Figure 3L–N). Collectively, these findings indicate that TG improved colonic barrier‐associated features and attenuated local inflammatory alterations in AAV‐SynA53T mice.

**FIGURE 3:**
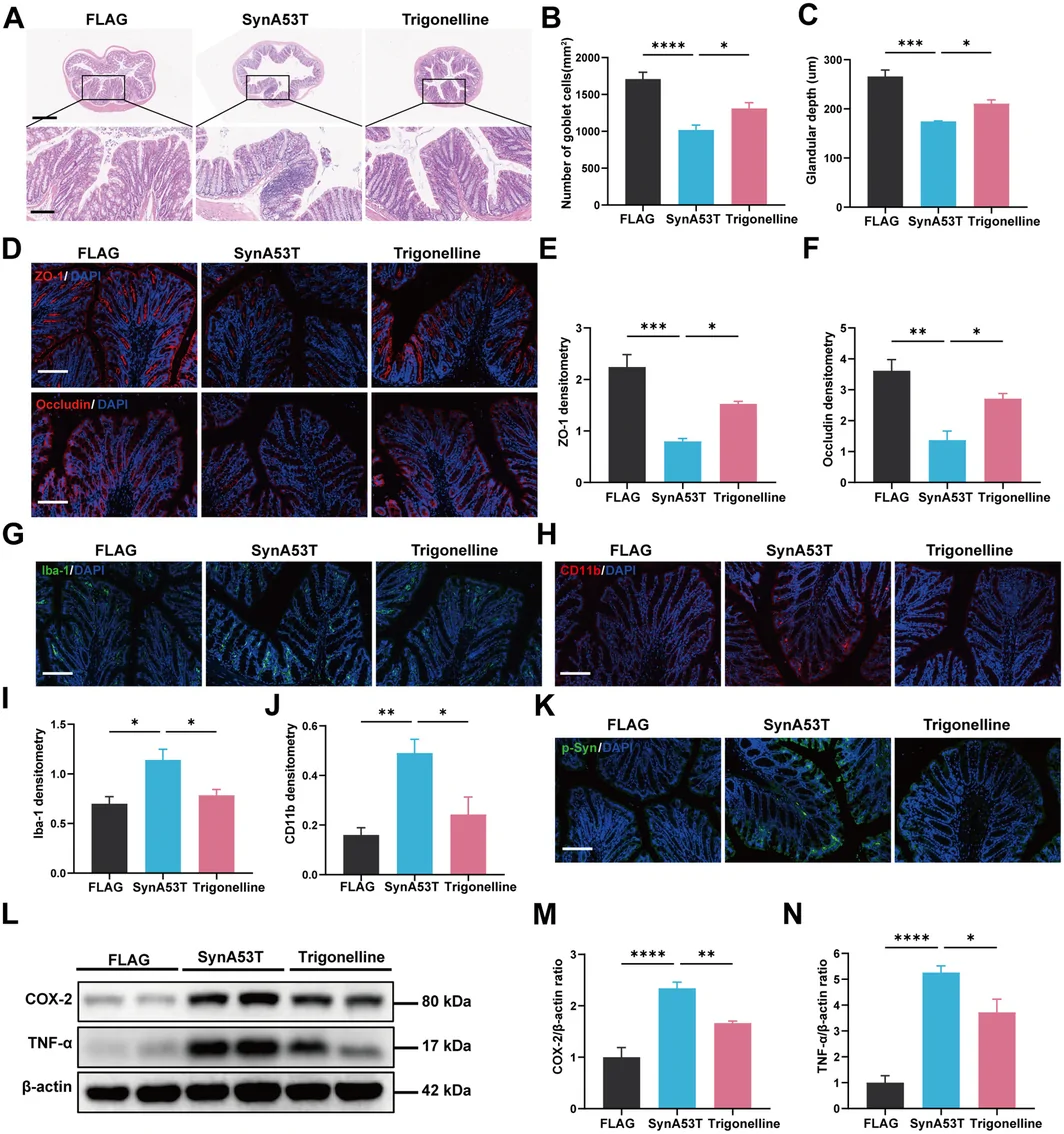
Trigonelline restores colonic architecture and suppresses inflammation and α‐synuclein pathology. (A) Representative H&E images of the colon. Scale bars: 500 μm (Upper panels); 100 μm (lower panels). (B) Quantification of goblet cell number (cells/mm^2^). (C) Quantification of glandular depth (μm). (D) Representative immunofluorescence staining of the tight‐junction proteins ZO‐1 and Occludin in the colon, with DAPI counterstaining. Scale bar: 100 μm. (E, F) Quantification of ZO‐1 and Occludin fluorescence densitometry. (G, H) Representative immunofluorescence staining of Iba‐1 and CD11b in the colon, with DAPI counterstaining. Scale bar: 100 μm. (I, J) Quantification of Iba‐1 and CD11b fluorescence densitometry. (K) Representative immunofluorescence staining of phosphorylated α‐synuclein (p‐α‐Syn) in the colon, with DAPI counterstaining. Scale bar: 100 μm. (L) Representative Western blots of COX‐2 and TNF‐α in colon tissue, with β‐Actin as a loading control. (M‐N) Quantification of COX‐2 and TNF‐α normalized to β‐Actin. Data are presented as mean ± SEM. Statistical significance: *p < 0.05, **p < 0.01, ***p < 0.001, ****p < 0.0001. H&E, hematoxylin and eosin; Iba‐1, ionized calcium‐binding adapter molecule 1; p‐α‐Syn, phosphorylated α‐synuclein; ZO‐1, zonula occludens‐1.

### 3.5 Trigonelline Reverses Gut Microbiota Dysbiosis and Reshapes the Microbial Community

Considering the critical role of the gut‐brain axis in Parkinsonian pathology, we investigated the restorative effects of TG on gut microbiota composition in AAV‐SynA53T mice using 16S rRNA sequencing. TG treatment significantly ameliorated the microbial disequilibrium observed in the disease model. The microbial dysbiosis index, which was elevated in SynA53T mice, was markedly reduced following TG administration (Figure 4A). This structural shift was further corroborated by Principal Coordinate Analysis (PCoA) based on Bray‐Curtis distances, which demonstrated a clear and significant separation between the microbiota of SynA53T and TG groups (PERMANOVA *R* = 0.7971, *p* = 0.001; Figure 4B), indicating a distinct reshaping of the global microbial landscape. Taxonomic profiling and biomarker analysis revealed specific alterations at the family and genus levels. While SynA53T control mice exhibited a dominance of potential pathobionts, TG treatment restored a more homeostatic profile. At the family level, TG increased the relative abundance of *Peptostreptococcaceae* and *Rikenellaceae* while reducing *Lactobacillaceae* (Figure 4C). LEfSe analysis (LDA score > 2.5) identified specific microbial signatures driving these differences: the SynA53T group was enriched with pro‐inflammatory or opportunistic taxa, including *Desulfovibrionaceae*, *Helicobacteraceae*, and the genera *Dubosiella* and *Desulfovibrio* (Figure 4E). Conversely, TG treatment significantly enriched beneficial taxa such as *Erysipelotrichaceae*, *Clostridiaceae*, and the genera *Ileibacterium*, *Bilophila*, and *Gordonibacter* (Figure 4E). To link these microbial shifts to phenotypic outcomes, we performed Spearman correlation analyses between specific bacterial taxa and motor performance metrics (Figure 4F,G). The abundance of *Desulfovibrionaceae* and *Eggerthellaceae* (at the family level) and *Desulfovibrio* (at the genus level) showed a significant positive correlation with the time required to complete the Beam and Pole tests, indicating an association with worsened motor function (Figure 4F,G). In contrast, the abundance of *Ileibacterium*, which was enriched by TG, displayed a significant negative correlation with test latencies, suggesting that the expansion of this genus is associated with improved motor performance (Figure 4G). Collectively, these data suggest that TG exerts its neuroprotective effects in part by suppressing PD‐associated pathobionts like *Desulfovibrio* and promoting beneficial commensals.

**FIGURE 4:**
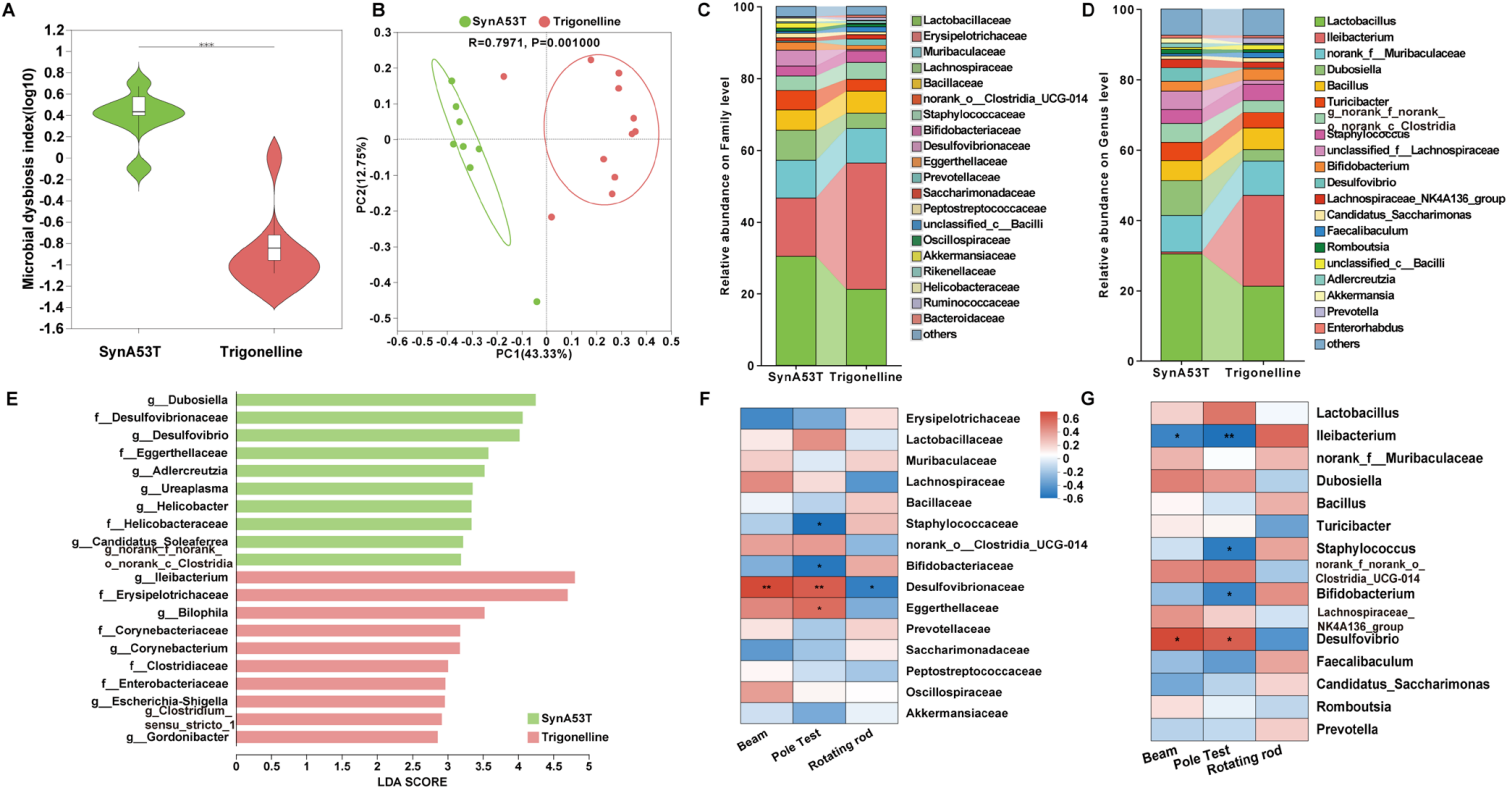
Trigonelline remodels gut microbiota composition. (A) Microbial dysbiosis index showing significantly reduced dysbiosis in TG treated mice. (B) Principal coordinate analysis (PCoA) based on Bray–Curtis distances demonstrating significant β‐diversity differences between groups (PERMANOVA *p* = 0.001). (C) Family‐level relative abundance comparison of top 20 taxa between SynA53T and TG mice. (D) Genus‐level relative abundance comparison of top 20 taxa. (E) Linear discriminant analysis effect size (LEfSe) identifying group‐specific taxa (LDA score > 2.5). (F) Correlation heatmap between behavioral parameters (rotarod latency, pole descent time, and beam across time) and microbial families. (G) Correlation heatmap between behavioral parameters and microbial genera. Positive correlations are shown in red; negative correlations in blue. Statistical significance annotated where applicable (*p* < 0.05). PCoA, principal coordinate analysis; LEfSe, Linear Discriminant Analysis Effect Size; LDA, Linear Discriminant Analysis; PD, Parkinson's disease.

### 3.6 Trigonelline Modulates the Gut Metabolome and Establishes Multi‐Omic Links With Host Behavior

With the aim of elucidating the functional consequences of TG‐induced microbiota remodeling, we performed untargeted LC–MS/MS metabolomic profiling of colon contents. Principal Component Analysis (PCA) revealed a distinct separation in the metabolic landscape between the TG group and the SynA53T group (Figure 5B). Using strict screening criteria (VIP > 1 and *p* < 0.05), we identified 309 differential metabolites, comprising 134 upregulated and 175 downregulated features (Figure 5A). To evaluate the concordance between TG‐associated microbial and metabolic alterations, we employed Procrustes analysis. The results demonstrated an evident concordance between the metabolomic configuration and the 16S‐derived microbial structure (Figure 5C), suggesting a coordinated relationship between TG‐associated microbiota remodeling and changes in the colonic metabolic environment. KEGG topology analysis further indicated that these differential metabolites were primarily enriched in pathways critical for lipid homeostasis and neuroprotection, including glycerophospholipid metabolism, taurine and hypotaurine metabolism, and alanine, aspartate, and glutamate metabolism (Figure 5D).

**FIGURE 5:**
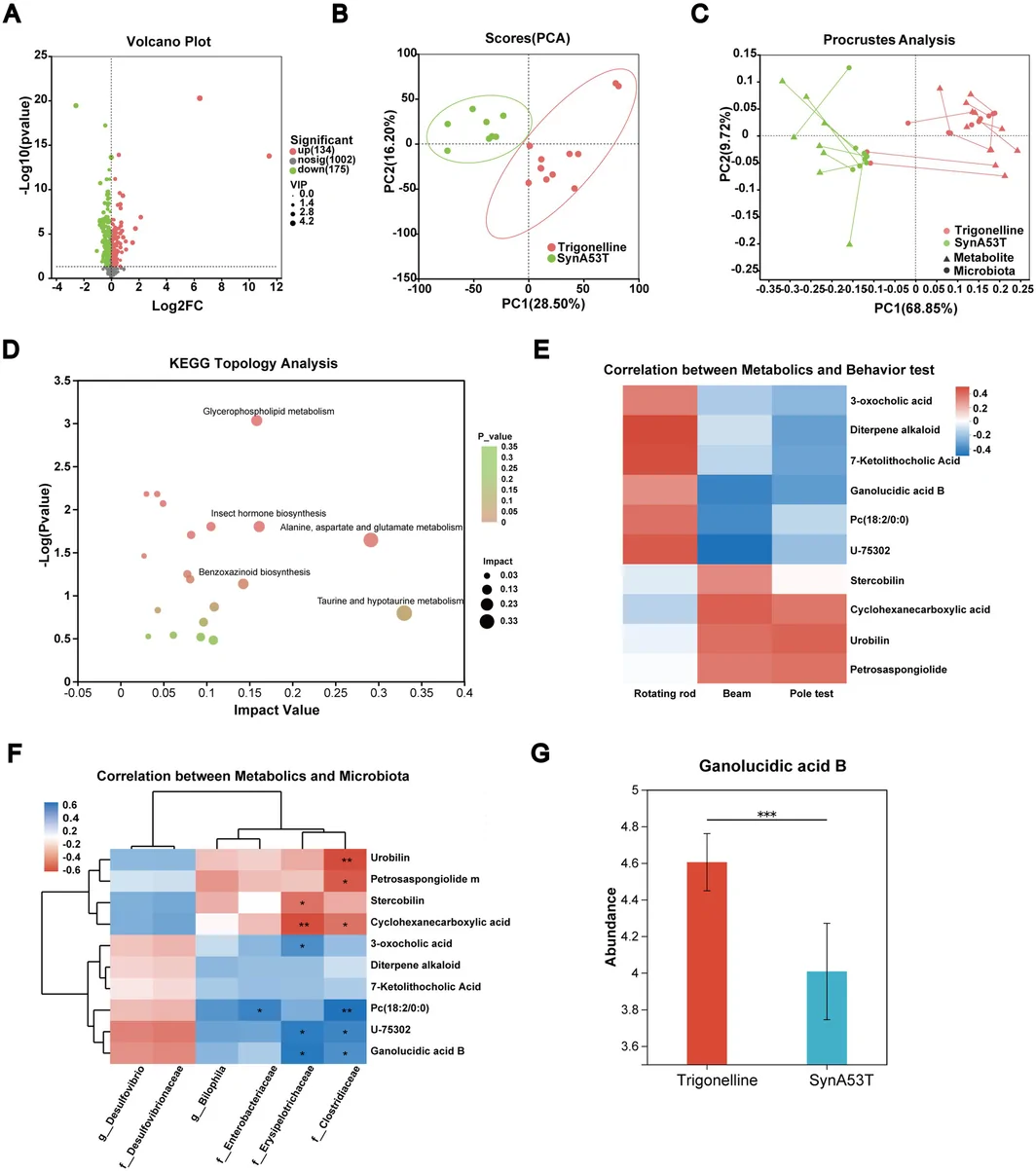
Metabolomics profiling and integrative analyses. (A) Volcano plot showing differential metabolites between the SynA53T and TG groups. (B) PCA score plot demonstrating separation of metabolic profiles between groups. (C) Procrustes analysis indicating concordance between metabolome and microbiome ordinations. (D) KEGG pathway topology analysis of differential metabolites (bubble size indicates pathway impact; color indicates significance). (E) Heatmap showing correlations between selected differential metabolites and behavioral performance. (F) Heatmap depicting correlations between representative metabolites and gut microbiota taxa. (G) Relative abundance of the metabolomic feature tentatively annotated as Ganolucidic acid B in the TG and SynA53T groups. Data are presented as mean ± SEM. Statistical significance: *p < 0.05, **p < 0.01, ***p < 0.001. PCA, principal component analysis.

We next integrated multi‐omics data with behavioral phenotypes to identify specific metabolites linking the gut microbiota to motor improvement. Spearman correlation analysis revealed a cluster of “beneficial” metabolites—notably 3‐oxocholic acid, 7‐ketolithocholic acid, and Ganolucidic acid B‐that exhibited a positive correlation with Rotarod performance (indicating better coordination) and a negative correlation with traversal time in the Beam and Pole tests (Figure 5E). Conversely, metabolites such as cyclohexanecarboxylic acid and urobilin showed the opposite pattern, associating with poorer motor outcomes. Among the metabolomic features associated with improved behavioral performance, the feature tentatively annotated as Ganolucidic acid B was prioritized for exploratory follow‐up based on its differential abundance and correlation patterns. Its abundance was significantly increased following TG treatment (Figure 5G). Furthermore, integrative analysis revealed that Ganolucidic acid B was positively correlated with TG‐ralated bacterial taxa (e.g., *Erysipelotrichaceae*, *Clostridiaceae*) and negatively correlated with taxa associated with the disease phenotype (e.g., *Desulfovibrio*, *Desulfovibrionaceae*) (Figure 5F). Based on this convergence of behavioral, microbial, and metabolomic evidence, Ganolucidic acid B was prioritized as a candidate TG‐associated metabolite for subsequent exploratory network pharmacology analysis.

### 3.7 Conditional Exploratory Network Analysis Based on the Feature Tentatively Annotated as Ganolucidic Acid B

To explore the potential molecular relevance of Ganolucidic acid B to Parkinson's disease, we integrated predicted compound targets with PD‐associated genes using a network pharmacology workflow. By intersecting the predicted targets of Ganolucidic acid B with known PD‐associated genes, we identified 146 overlapping targets (Figure 6A). These common targets were mapped into a protein–protein interaction (PPI) network, which showed dense connectivity among the included nodes (Figure 6B). However, network connectivity should not be interpreted as evidence of functional synergy or target engagement. To screen for highly connected predicted nodes within this network, we applied three topological algorithms: Maximal Clique Centrality (MCC), Maximum Neighborhood Component (MNC), and Degree (Figure 6C–E). The intersection of the top‐ranked candidates from these algorithms yielded 10 consensus hub genes (Figure 6F). Notably, this core cluster included key components of the PI3K–AKT signaling cascade (PIK3CA, PIK3CD, PIK3R1) alongside prominent inflammatory and transcriptional regulators (NFKB1, STAT3, TNF, MAPK3), forming a tightly interconnected regulatory module (Figure 6G). Further cluster analysis extracted key subnetworks centered around these hubs (Figure 6H,I), suggesting that the predicted targets of Ganolucidic acid B may be associated with these inflammatory and survival‐related nodes. We next conducted functional enrichment analyses to elucidate the biological processes driven by these targets. Gene Ontology (GO) analysis revealed a significant enrichment in inflammation‐related processes, specifically “inflammatory response” and “negative regulation of inflammatory response” (Figure 6J), suggesting a potential association with immunomodulatory processes. Crucially, Kyoto Encyclopedia of Genes and Genomes (KEGG) pathway analysis prioritized the PI3K–Akt signaling pathway as the most enriched pathway (highest gene count), followed closely by inflammation‐associated pathways such as TNF, Chemokine, and JAK–STAT signaling (Figure 6K). These exploratory network pharmacology data suggest that predicted targets of Ganolucidic acid B are enriched in PD‐relevant inflammatory and PI3K–AKT‐related pathways. However, these computational predictions do not establish direct target engagement or functional pathway inhibition.

**FIGURE 6:**
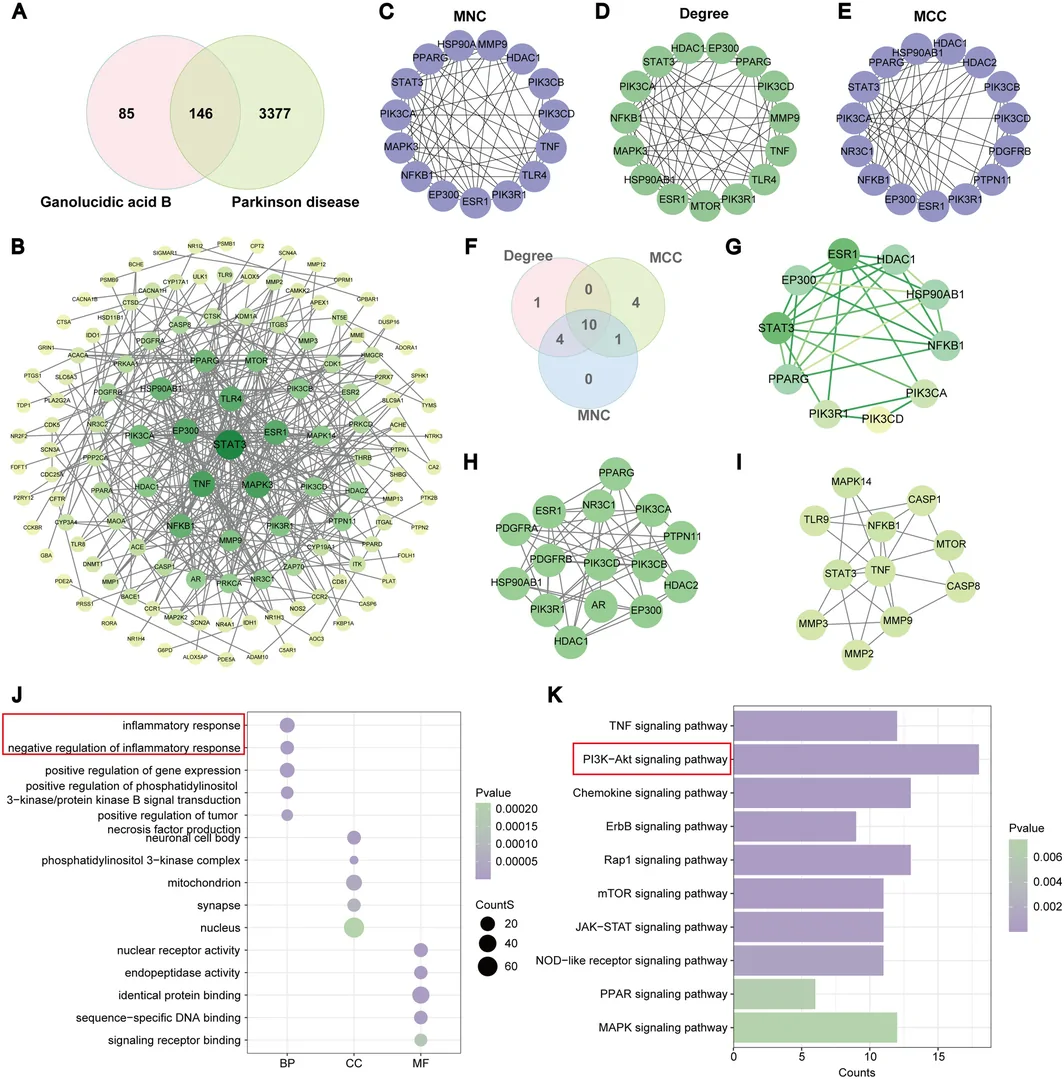
Conditional exploratory network analysis based on the feature tentatively annotated as Ganolucidic acid B. (A) Venn diagram showing the overlap between predicted targets of Ganolucidic acid B and PD‐related genes from GeneCards. (B) PPI network of the 146 intersecting genes. (C–E) Topological analyses of the PPI network identifying candidate hub genes using the MNC, Degree, and MCC algorithms, respectively. (F) Venn diagram summarizing the overlap of hub genes identified by the three algorithms. (G) Core hub‐gene network derived from the consensus set. (H, I) MCODE module analysis showing major functional clusters within the PPI network. (J, K) GO functional annotation and KEGG pathway enrichment analyses of the intersecting targets. PD, Parkinson's disease; PPI, protein–protein interaction; MCC, Maximal Clique Centrality; MNC, Maximum Neighborhood Component; GO, Gene Ontology; KEGG, Kyoto Encyclopedia of Genes and Genomes.

## 4 Discussion

TG improved motor performance, preserved dopaminergic markers, and reduced α‐synuclein pathology and inflammation. These changes were accompanied by reduced PI3K/AKT–NF‐κB signaling activity, improved intestinal barrier‐associated features, and altered gut microbial and metabolic profiles. Collectively, our data support TG as a modulator of both central and peripheral pathological changes in AAV‐SynA53T mice.

Coffee contains multiple bioactive constituents, among which TG has comparatively stronger evidence in neurodegeneration research. For example, oral TG improved cognition‐related behaviors in Alzheimer's disease (AD) model mice and was detectable in the brain, with mechanistic work implicating processes related to neurite outgrowth (Farid et al. 2020). Recent mechanistic reviews further summarize TG as a candidate with antioxidant, anti‐inflammatory, and metabolic homeostasis–regulating potential (Liang et al. 2023). Our MR analysis provided supportive genetic evidence for an inverse association between genetically predicted serum TG and α‐synuclein levels, subject to the assumptions of MR (Sanderson et al. 2022). Moreover, in an AAV‐SynA53T model, we observed that TG improved motor performance and reduced pathological pSer129‐α‐syn signals in the midbrain. These findings align with prior studies linking coffee consumption—a primary source of trigonelline—to reduced PD risk (Kuzovenkova et al. 2025; Zhao et al. 2024). The translational relevance of the dose used in this study should also be considered. The 5 mg/kg/day oral dose was selected based on previous mouse work using the same TG dose and treatment duration (Aktar et al. 2024). After body surface area normalization, this dose corresponds approximately to 24.3 mg/day for a 60 kg adult, which overlaps with the reported range of TG content in some coffee preparations (Angelino et al. 2018). Thus, the present dose appears broadly compatible with a dietary‐exposure range rather than a clearly supraphysiological pharmacological dose, although direct equivalence to a defined number of coffee servings should be interpreted cautiously because TG intake varies with coffee type, roasting, preparation method, serving size, absorption, and metabolism.

To further clarify how TG works, we examined key inflammatory and survival signaling pathways implicated in PD. In the substantia nigra of AAV‐SynA53T mice, we observed marked activation of the NF‐κB signaling pathway, evidenced by increased phosphorylation of NF‐κB p65 and elevated expression of downstream pro‐inflammatory mediators including TNF‐α. TG administration substantially attenuated this inflammatory response, reducing both TNF‐α levels and NF‐κB p65 phosphorylation. Notably, existing pharmacological evidence highlights TNF‐α/NF‐κB signaling as a pivotal therapeutic target in PD, where its inhibition effectively reduces microglial activation and dopaminergic degeneration (Chong et al. 2014; He et al. 2025; Zhang et al. 2016). These findings align with recent studies confirming that neuroinflammation drives PD progression through microglial overactivation and TNF‐α‐mediated damage (Harms et al. 2021; Imamura et al. 2003), and are consistent with previous research demonstrating the effect of TG to suppress NF‐κB signaling in the brain under inflammatory conditions (Khalili et al. 2018). The involvement of PI3K/AKT signaling suggests a regulatory upstream mechanism. PD mice often exhibit dysregulated PI3K/AKT signaling in microglia and neurons (Isik et al. 2023; Kim et al. 2023; Wang et al. 2024). In our study, TG was also found to ameliorate microglial polarization abnormalities caused by dysregulated PI3K/AKT pathway activation. Recent reviews further emphasize that modulating NF‐κB signaling downstream of AKT in microglia represents a promising therapeutic strategy for counteracting sustained neuroinflammation in neurodegenerative diseases (Chu et al. 2021). Together, these findings suggest that the protective effects of TG are associated with reduced PI3K/AKT‐NF‐κB signaling activity and TNF‐α expression, alongside attenuated microglial activation.

Increasing evidence indicates that PD involves substantial peripheral and intestinal pathological changes, including gut barrier dysfunction, intestinal inflammation, gut microbial dysbiosis, and microbial–metabolic alterations (Liao et al. 2024; Sharma et al. 2019; Wallen et al. 2022). Therefore, we further examined whether TG also exerted protective effects on intestinal pathology in AAV‐SynA53T mice. TG treatment effectively restored colonic tight‐junction integrity and mucosal architecture, supporting improved barrier‐associated features and potentially reducing pro‐inflammatory peripheral cues. This improvement of gut barrier function is a critical mechanism through which dietary intake of bioactive compounds can exert systemic benefits (Li et al. 2024); however, their mediating role was not tested in the present study. Beyond this, TG demonstrated significant immunomodulatory activity within the intestinal environment. In AAV‐SynA53T mice, we observed substantial inflammation marked by elevated infiltration of Iba‐1^+^ and CD11b^+^ immune cells in the colon, along with increased expression of pro‐inflammatory mediators including TNF‐α and COX‐2. These observations are consistent with established literature documenting that intestinal inflammation in PD involves heightened activation of macrophage and microglia‐like populations, which contribute to localized cytokine production and impaired epithelial barrier function (Mulak and Bonaz 2015; Munoz‐Pinto et al. 2024). TG treatment significantly attenuated these responses. The anti‐inflammatory effect is supported by prior research showing that TG attenuates intestinal inflammation through downregulation of pro‐inflammatory mediators including TNF‐α and IL‐1β while enhancing antioxidant capacity in experimental colitis models (Omidi‐Ardali et al. 2019). However, previous evidence showing that orally administered TG can be detected in plasma and cerebral cortex suggests that TG may also exert direct effects within the CNS (Farid et al. 2020). Therefore, the intestinal improvements observed in this study may coincide with the central neuroprotective effects of TG and may partially mediate these effects, yet our findings do not confirm that TG exerts its neuroprotective function primarily or solely through a gut–brain axis‐dependent mechanism.

Colon content 16S rRNA sequencing revealed that TG significantly remodeled the gut microbiota structure in PD mice, selectively promoting beneficial taxa while suppressing genera overrepresented in disease models. A cohort study demonstrated depleted *Erysipelotrichaceae* in PD patients, showing consistency with our finding (Bai et al. 2024). TG increased the abundance of *Erysipelotrichaceae* relative to the SynA53T group. Members of this family have been linked to immune and metabolic regulation and intestinal inflammation (Kaakoush 2015). The enrichment of *Desulfovibrio* observed in rotenone‐induced PD models also aligns with our 16S rRNA sequencing data (Zhao et al. 2021). TG further modulated PD‐relevant pathobionts by suppressing *Desulfovibrio*, a genus increasingly linked to PD, as patient‐derived *Desulfovibrio* strains can enhance α‐synuclein aggregation in vivo and may thereby facilitate enteric α‐syn pathology with potential propagation to the brain (Huynh et al. 2023). Our correlation analyses also revealed negative associations between *Desulfovibrio* abundance and motor performance. Notably, plasma metabolomic evidence also suggests that circulating TG levels can be microbiota‐regulated, supporting a feed‐forward scenario in which dietary TG reshapes the microbiota and, in turn, microbial remodeling influences the availability and activity of food‐derived neuroprotective components (Manghi et al. 2024).

The integration of metabolomics and 16S rRNA sequencing identified a metabolomic feature tentatively annotated as Ganolucidic acid B that was associated with several taxa enriched in the TG group and with behavioral measures. This feature was therefore selected for a conditional, hypothesis‐generating network analysis. Because its chemical identity was not confirmed using an authentic standard, the resulting target and pathway predictions should be interpreted solely as exploratory and conditional on the tentative annotation.

Several limitations should be acknowledged. First, although the AAV‐SynA53T model recapitulates key PD‐like features, including α‐synuclein pathology, dopaminergic impairment, neuroinflammatory activation, and motor deficits, it cannot fully reproduce the chronic, multifactorial, and heterogeneous nature of human PD. Thus, the observed therapeutic effects of TG should be interpreted as preclinical evidence in an α‐synucleinopathy‐related model rather than direct evidence of clinical efficacy. Second, the MR analysis should be interpreted cautiously. Its validity depends on assumptions regarding instrument strength, independence, and exclusion restriction, and may be affected by weak instrument bias, horizontal pleiotropy, population heterogeneity, and differences between genetically predicted circulating TG and experimentally administered TG. Therefore, the MR results should be viewed as supportive genetic evidence rather than standalone proof of the biological mechanism observed in mice. Third, the observed changes in PI3K/AKT–NF‐κB signaling remain correlative and do not establish pathway dependency. Similarly, although TG treatment was accompanied by alterations in gut microbiota composition, intestinal barrier‐associated markers, and colonic metabolomic profiles, these multi‐omics findings reflect system‐level associations rather than direct causal evidence along the gut–brain axis. Therefore, the present data cannot determine whether gut‐associated changes are required for TG‐mediated neuroprotection or whether they occur in parallel with direct CNS‐related effects. Finally, Ganolucidic acid B was prioritized based on untargeted metabolomic annotation and correlation analysis. Without authentic standard‐based validation, high‐confidence MS/MS confirmation, targeted quantification, and functional assays, it should be regarded as a candidate TG‐associated metabolite rather than a confirmed functional mediator.

In conclusion, TG ameliorates motor deficits, α‐synuclein pathology and neuroinflammation in AAV‐SynA53T mice. These effects are associated with regulation of PI3K/AKT and NF‐κB signaling, as well as remodeling of gut microbiota and metabolic profiles. Ganolucidic acid B is identified as a candidate metabolite related to TG treatment, while its exact structure and functional role require further validation. Collectively, these findings suggest that TG holds promise as a nutritional neuroprotective agent and provide a basis for subsequent studies on its direct central effects, gut‐mediated mechanisms and metabolite‐specific actions.

## Author Contributions

**Mozi Chen:** writing – original draft, writing – review and editing, project administration. **Yiyao Dong:** conceptualization, methodology, formal analysis, investigation, data curation. **Jie Zheng:** formal analysis, data curation. **Yan Lin:** software, data curation. **Qiyao Li:** writing – original draft. **Chuhua Wei:** investigation. **Linpei Zhuo:** software. **Yan He:** data curation. **Jing Sun:** writing – review and editing, formal analysis. **Feng Fu:** project administration. **Jiaming Liu:** writing – original draft, writing – review and editing, project administration.

## Funding

This research was supported by the Projects of Administration of Traditional Chinese Medicine of Zhejiang Province (No. 2025ZL140).

## Ethics Statement

All animal experimental procedures were approved by the Animal Ethics Committee of Wenzhou Medical University (No. wydw2025‐0555) and adhered to the National Institutes of Health Guide for the care and use of laboratory animals. No human subjects were involved in the present study, so consent to participate is deemed inapplicable.

## Consent

The authors have nothing to report.

## Conflicts of Interest

The authors declare no conflicts of interest.

## Supporting information


**Figure S1:** The relationship between trigonelline level on α‐synuclein using MR analysis. (A) Forest plot of MR analyses for the causal effects of TG on α‐synuclein. (B) The scatter plot of 8 SNPs applied 5 MR methods; (C) The leave‐one‐out sensitivity analysis plot for TG on α‐synuclein. MR, Mendelian randomization; SNP, single nucleotide polymorphism.

**Table S1:** SNPs used as instrumental variables and corresponding F‐statistics in the Mendelian randomization analysis.
**Table S2:** RT‐qPCR primer sequence.

## Data Availability

The datasets used and analyzed during the current study are available from the corresponding author on reasonable request.
