# Supplementary material for: Trigonelline Alleviates Neuroinflammation in AAV‐SynA53T Parkinson's Disease Model Mice: Involvement of PI3K/AKT–NF‐κB Signaling and Gut Microbial‐Metabolic Remodeling
Source: Food Sci Nutr. 2026 Oct 6;14(10):e72451. doi: 10.1002/fsn3.72451 (PMC13643061; doi:10.1002/fsn3.72451)
Supplement: Supplementary file 1 [file FSN3-14-e72451-s002.docx]

| **Table S1** **SNPs used as instrumental variables and corresponding F-statistics in the Mendelian randomization analysis**. | |
| --- | --- |
| **SNP** | **F-statistic** |
| rs12756028 | 28.20101153 |
| rs78947754 | 22.12162174 |
| rs77372529 | 21.73139457 |
| rs73156540 | 24.27351538 |
| rs143034895 | 21.78349294 |
| rs62078170 | 23.56740503 |
| rs147681404 | 20.86514145 |
| rs5992074 | 22.65268234 |
| Note: F-statistics were calculated as beta^2^/SE^2^ based on the exposure GWAS for serum trigonelline. F > 10 was considered indicative of sufficient instrument strength. | |

| **Table S2** **RT-qPCR primer sequence** | | |
| --- | --- | --- |
| Gene | Sequence (5'-3') | |
| Actin | Forward: | GGCTGTATTCCCCTCCATCG |
|  | Reverse: | CCAGTTGGTAACAATGCCATGT |
| IL-1β | Forward: | GCAACTGTTCCTGAACTCAACT |
|  | Reverse: | ATCTTTTGGGGTCCGTCAACT |
| IL-6 | Forward: | CCAAGAGGTGAGTGCTTCCC |
|  | Reverse: | CTGTTGTTCAGACTCTCTCCCT |
| NFkb1 | Forward: | ATGGCAGACGATGATCCCTAC |
|  | Reverse: | TGTTGACAGTGGTATTTCTGGTG |
